# Toxicity, Deterrent and Repellent Activities of Four Essential Oils on *Aphis punicae* (Hemiptera: Aphididae)

**DOI:** 10.3390/plants11030463

**Published:** 2022-02-08

**Authors:** Samy Sayed, Mohamed Mohamed Soliman, Saad Al-Otaibi, Mohamed M. Hassan, Sayed-Ashraf Elarrnaouty, Samia M. Abozeid, Ahmed M. El-Shehawi

**Affiliations:** 1 Department of Science and Technology, University College-Ranyah, Taif University, Taif 21944, Saudi Arabia; 2 Clinical Laboratory Sciences Department, Turabah University College, Taif University, Taif 21995, Saudi Arabia; mmsoliman@tu.edu.sa; 3 Department of Biotechnology, College of Science, Taif University, Taif 21944, Saudi Arabia; dralotaiba@yahoo.com (S.A.-O.); a.elshehawi@tu.edu.sa (A.M.E.-S.); 4 Department of Biology, College of Science, Taif University, Taif 21944, Saudi Arabia; m.khyate@tu.edu.sa; 5 Department of Economic Entomology and Pesticides, Faculty of Agriculture, Cairo University, Giza 12613, Egypt; ashrafelarnaouty@agr.cu.edu.eg; 6 Plant Protection Research Institute, Agricultural Research Center, Dokki, Giza 12618, Egypt; drsamiamonzer43@gmail.com

**Keywords:** aromatic plants, Lamiaceae family, essential oils, biological control, aphids, insect predators

## Abstract

Chemical insecticides have many harmful effects, including as foodborne residues and environmental contaminants, as well as side effects on natural enemies and serious risks for human health. The use of plant-derived essential oils (EOs) as effective bio-agents has become an essential component of integrated pest management. In this study, the contact toxicity, deterrent, and repellent activities were evaluated for essential oils obtained from *Mentha piperita, Mentha longifolia, Salvia officinalis*, and *Salvia rosmarinus*, grown at high altitudes in the Taif region, KSA, on *Aphis punicae*. Furthermore, the toxicity of these EOs against the predator *Coccinella undecimpunctata* was estimated. A total of 17, 14, 16, and 26 compounds were identified in the EOs of *M. piperita*, *M. longifolia*, *S. officinalis*, and *S. rosmarinus*, respectively. They showed a variation in the major compounds: *M. piperita* (Carvone, 61.16%), *M. longifolia* (Pulegone, 48.6%), *S. officinalis* (Eucalyptol, 33.52%), and *S. rosmarinus* (α-pinene, 36.65%). A contact toxicity test on *A. punicae* imago and *C. undecimpunctata* larvae showed that LC_50_ were approximately four-fold greater for all tested EOs towards aphids compared to towards the predator, while the two species of *Salvia* sp. were more effective than the other two species of *Mentha* sp. The LC_50_ values on *A. punicae* ranged from 1.57 to 2.97 µg/mL, while on *C. undecimpunctata* larvae, they ranged from 5.96 to 10.33 µg/mL. Furthermore, the EOs of two species of *Salvia* sp. showed excellent repellence and deterrence against *A. punicae*. In conclusion, the tested EOs, especially those from *Salvia* sp., have been shown to be promising natural aphicides, repellent, and deterrent against *A. punicae*, and they are safe for important insect predators.

## 1. Introduction

The widespread utilization of synthetic pesticides poses hazards for both the environment and human health due to their toxicity and poor biodegradability [1,2]. In addition, the use of some chemical pesticides or their residues may be hazardous for non-target organisms, including humans and beneficial organisms [3,4]. Therefore, farmers need alternative and safe agricultural methods, including the use of natural products, to achieve more sustainable production strategies. Recently, in plant protection, there has been a growing interest in botanical pesticides, which contain active ingredients composed of natural compounds such as essential oils (EOs) [5,6].

Essential oils (EOs) derived from medicinal and aromatic plants are considered safe substances for the environment and human health. Thus, they can be used as active substances for pest control [7]. In this regard, many investigations have stated the potential of EOs as natural pesticides for integrated pest management (IPM) [1]. EOs derived from different plants exhibit unique medicinal and botanical activities that, upon suitable application, may not cause negative effects for animal and human health. The modes of action of EOs on pests include various methods, such as contact toxicity, repellent, antifeedant, fumigant, and growth-inhibiting activity [8]. The main benefit of botanical pesticides is that they provide residue-free food and a safe environment. Moreover, they affect only target insects and do not have considerable negative effects on the beneficial insects such as pollinators and natural enemies [9]. Plant EOs are potentially valuable for pest control. They performed in different ways on various insect pests and can be applied to many crops or stored products [9,10]. EOs and their chemical constituents have considerable fumigant and contact toxicity towards numerous insect and mite pests and plant pathogenic fungi [6,11]. Moreover, EOs can be highly effective on pesticide-resistant insects; in addition, the use of chemical pesticides can create dangerous residues when used against insect pests on plants [12]. EOs are secondary metabolites that play an important role in protecting plants from herbivores or pathogens [13]. Generally, they are composed of complex mixtures of phenols, monoterpenes, and sesquiterpenes, and they have demonstrated antifeedant, insecticidal, repellent, deterrent, and insect growth regulation effects [8,11].

The pomegranate aphid *Aphis punicae* Passerini (Hemiptera: Aphididae) is the main sucking–piercing insect pest affecting pomegranate. Both nymphs and imagos infest young leaves, vegetative and flower buds, flowers, and young fruits, resulting in the discoloration and drying of these affected parts [13,14]. Moreover, they secrete honeydew, which causes sooty molds, therefore inhibiting photosynthesis and causing a remarkable loss in quality and quantity of the crop yield [15].

Eos contain various volatiles, low-molecular-weight phenolics, and terpenes. The major families of plants from which EOs are extracted include Lamiaceae, Myrtaceae, Asteraceae, and Lauraceae. Eos have insecticidal, repellent, and growth-reducing effects on various species of insects. They have been utilized viably to control preharvest and postharvest phytophagous insects [8].

The Lamiaceae family includes approximately 220 genera and 3300 species. The genus *Mentha* also belongs to the Lamiaceae family and includes more than 25 species. *Mentha piperita* and *M. longifolia*, commonly known as peppermint and wild mint, respectively, are frequently cultivated in many countries for the production of EOs [16,17]. *Salvia* is the largest genus of the Lamiaceae family, commonly known as sage, and consists of approximately 1000 species distributed in subtropical, tropical, and temperate regions all over the world [18]. Rosemary (*Rosmarinus officinalis* L.) belongs to the Lamiaceae family. In a recent phylogenetic analysis, the genus *Rosmarinus* was merged into the genus *Salvia*. After this merging was done, the species *R. officinalis* became known under the name *Salvia rosmarinus* [19].

The season and the location have strong effects on the chemical composition of Eos obtained from the same plant organ, especially the leaves. Therefore, the biological properties, such as insecticidal, antioxidant, and anti-inflammatory activity, are variable [20]. In the current study, we aimed to extract EOs from four species of aromatic plants—*Mentha piperita, Mentha longifolia, Salvia officinalis,* and *Salvia Rosmarinus*—grown at high altitudes in the Taif region, KSA, and to evaluate the aphicidal, deterrent, and repellent activities of the EOs against *A. punicae*. Due to the importance of evaluating the effects of insecticides on the important natural enemies of insect pests, the toxicity of these EOs against *Coccinella undecimpunctata* L. (Coleoptera: Coccinellidae) was also estimated where this species is common in this area of the study [21], and it was also noticed on the pomegranate trees during this study.

## 2. Materials and Methods

### 2.1. Plant Material and Essential Oil Extraction

One kg of fresh leaves of *M. x piperita, M. longifolia, S. officinalis,* and *S. rosmarinus* were obtained from the Al-Hada region (21°21′32.98″ N and 40°17′15.08″ E), Taif Governorate, Saudi Arabia, which is considered a high-altitude region (2000 m above sea level). Leaves were air-dried and ground to a fine powder. Then, 100 g of the powder from each plant was used as a replicate (3 replicates) to extract the essential oils. Each 33.3 g was inserted into a 1-L flask filled with 0.5 L of distilled water and subjected to hydrodistillation using a Clevenger-type apparatus for six hours [22]. The oils were dried over anhydrous sodium sulfate to remove traces of moisture and stored at 4 °C until use.

### 2.2. Insects

Some leaves of pomegranate infested with *A. punicae* were collected from the field, then, they were transferred to 10 pomegranate seedlings cultivated in a greenhouse for their mass rearing in order to use them in the experiments. Then, infested leaves were transferred daily to the laboratory to remove adults and keep nymphs only in order to collect new adults (1 day old) the next day (the day of the experiment beginning). Adults and larvae of the predator, *C. undecimpunctata,* were also collected from the same plants and transferred to the laboratory. They were fed on eggs of *Ephestia kuehniella* (Zeller) (Lepidoptera: Pyralidae) to obtain sufficient individuals for the experiments. 

### 2.3. GC–MS Analysis of Essential Oils

The GC–MS analysis was carried out using a gas chromatography–mass spectrometry instrument at the Department of Medicinal and Aromatic Plants Research, National Research Center, with the following specifications. Instrument: a TRACE GC Ultra Gas Chromatograph (THERMO Scientific Corp., Waltham, MA, USA), coupled with a THERMO mass spectrometer detector (ISQ Single Quadrupole Mass Spectrometer), Waltham, MA, USA. The GC–MS system was equipped with a TG-WAX MS column (30 m × 0.25 mm i.d., 0.25 μm film thickness), Waltman, MA, USA. Analyses were performed using helium as a carrier gas at a flow rate of 1.0 mL/min and a split ratio of 1:10 with a temperature program as follows: 60 °C for 1 min; rising at 3.0 °C /min to 240 °C and held for 1 min. Both the injector and detector were held at 240 °C. A diluted sample (1:50 hexane, v/v) of 1 μL of the mixture was injected. The mass spectrum was obtained by electron ionization (EI) at 70 eV at a spectral range of m/z 40–450. Compounds were identified using the analytical method: mass spectra (authentic chemicals, Wiley spectral library collection and NSIT library).

### 2.4. Contact Toxicity

Essential oils were diluted with *n*-hexane at the following concentrations: 1, 3, 5, 10 and 20 µg/mL. Meanwhile, *n*-hexane alone was used as a control. The contact toxicities of the EOs were estimated by the leaf immersion method. Pomegranate leaves of approximately the same size (≈3 cm in long) were immersed in the tested concentrations for 3 s and then air-dried for 30 s. Twenty aphid imagos of *A. punicae* (1 day in age) were transferred with a brush from the plant leaves of the rearing colony to the treated leaves in Petri dishes. Petri dishes were wrapped with parafilm to prevent the escape of aphids. Each treatment was replicated five times and mortality was recorded after 24 h. For the coccinellid, *C. undecimpunctata*, filter papers (Whatman No. 1) were used in this experiment. Filter papers (9 cm in diameter) were immersed for 3 s in the tested concentrations of EOs, where *n*-hexane was used as a control. Both treated and control papers were air-dried and placed into a Petri dish. Then, 20 of the 3rd larval instar were placed in each dish using a brush, with a sufficient amount of *Ephestia kuehniella* eggs as a food source. The dishes were placed in an incubator and the number of surviving and dead individuals was counted after 24 h on control and treated papers. 

### 2.5. Deterrent Test

This experiment is to estimate the nymph production deterrence of aphid imagos. Pomegranate leaves were used in this experiment, where some leaves were immersed in *n*-hexane (control), while others (treatments) were immersed for 3 s in one of the following estimated three lethal concentrations: LC_10_, LC_20_, and LC_30_, from the contact assay of each essential oil. Therefore, it could be possible to estimate if the live aphid adults were affected by the EOs or not in their nymph production. Both treated and control papers were air-dried for 30 s and each one was placed into a Petri dish. Then, 5 aphid imagos were transferred to each dish using a brush. Petri dishes were maintained with moistened filter paper to maintain the moisture; then, dishes were wrapped with parafilm to prevent the escape of aphids. Each treatment was carried out with five replicates. The dishes were placed in an incubator and the number of aphid individuals was counted after 3 days on control and treated leaves. Deterrence (%) was calculated for each dish as follows: 1 − (Nt − Nc) × 100, where C is the number of individuals on the control leaf and T is the number on the treated leaf [23].

### 2.6. Repellent Test

Filter papers (9 cm in diameter) were cut in half, where one half was dipped in *n*-hexane (control), while the other was dipped for 3 s in one of the following concentrations: 1, 3, 5, 10 and 20 µg/mL. Both treated and control papers were air-dried and placed into a Petri dish. Then, 20 aphids were placed in the center of each dish using a brush. Petri dishes were wrapped with parafilm to prevent the escape of aphids. Each treatment was carried out with five replicates. The dishes were placed in an incubator and the number of aphid individuals was counted after 12 h on control and treated papers. Repellence percentages were calculated according to the following formula: The percent repellence (PR) $\mathrm{PR}\left(\%\right)=\left[\frac{\mathrm{Nc}-\mathrm{Nt}}{\mathrm{Nc}+\mathrm{Nt}}\right]\times 100\;$ [24], where Nc is the number of individuals found in the negative control half and Nt is the number found in the treated half. 

### 2.7. Statistical Analysis 

The mortality (%) in each treatment was corrected with that in the control depending on Abbott’s formula. The selectivity ratios (LC50 for the predator/LC50 for aphid) were estimated [25]. Each lethal concentration (LC_50_) was estimated using Probit analysis. Then, significant differences among the LC_50_ values were determined using the confidence intervals of the relative median potency (RMP). Differences between each pair of LC_50_ values were considered statistically significant if 1.0 was not present in the 95% confidence interval of RMP. Analysis of variance (One-Way ANOVA) and Tukey’s test were conducted to assess repellent and deterrent effects. Moreover, Two-Way ANOVA was performed to estimate the interaction between EOs and concentrations on aphid deterrence. Statistical analysis was determined using the SPSS software program, version 20, Armonk, NY: IBM Corp, USA.

## 3. Results

### 3.1. Yields and Chemical Composition of Essential Oils

The obtained yields of EOs were 0.72 ± 0.03, 0.94 ± 0.09, 1.35 ± 0.08, and 0.89 ± 0.05 (w/w) for *M. x piperita, M. longifolia, S. officinalis,* and *S. rosmarinus,* respectively. The chemical compositions of the tested essential oils are presented in Table 1 and Figure S1. A total of 17, 14, 16, and 26 compounds were identified in the EOs of *M. piperita, M. longifolia, S. officinalis,* and *S. rosmarinus,* respectively. The major component of *M. piperita* EO was Carvone (61.16%), followed by α -Cubebene (10.99%) and D-Limonene (4.08%). In *M. longifolia*, Pulegone (48.6%), l-Menthone (34.49%), and Eucalyptol (4.5%) were the major components. In *Salvia* sp., Eucalyptol (33.52%), α-pinene (22.68%), and Camphene (14.44%) were the major components in *S. officinalis,* while α-pinene (36.65%) was the major component in *S. rosmarinus*, followed by p-Cymene (7.08%), Eucalyptol (6.91%), and Camphene (6.8%).

### 3.2. Effect of Contact Toxicity

The LC_50_ values of the four tested EOs on the aphid *A. punicae* indicated that the LC_50_ values for both *S. officinalis* (1.574 µg/mL) and *S. rosmarinus* (1.653 µg/mL) EOs were lower than those of both *M. piperita* (2.971 µg/mL) and *M. longifolia* (2.4 µg/mL) (Table 2). The same trend was obtained for *C. undecimpunctata* larvae, where the LC_50_ values were 6.237, 5.960, 10.334, and 8.737 µg/mL for *S. officinalis, S. rosmarinus, M. piperita,* and *M. longifolia,* respectively (Table 2). It was noticed that the selectivity ratios were ranged from 3.478 to 3.963. This means that the coccinellid predator is more tolerant for these EOs than the aphid species. The comparison between the LC_50_ values of each pair of EOs on *A. punicae* by RMP analyses indicated that all comparisons among the four tested EOs were non-significant, except for *M. piperita* versus *S. officinalis* (RMP = 1.705, 95% CI: 1.031, 2.433) and *S. officinalis* versus *S. rosmarinus* (RMP = −0.195, 95% CI: −0.871, 0.475). Meanwhile, these comparisons for *C. undecimpunctata* larvae showed that that all comparisons among the four tested EOs were significant, except for *M. longifolia* versus *M. piperita* (RMP = 0.933, 95% CI: 2.833) and *S. rosmarinus* versus *S. officinalis* (RMP = 0.756, 95% CI: −2.664, 0.475) (Table 3).

### 3.3. Deterrent Activity

**Figure 1:**
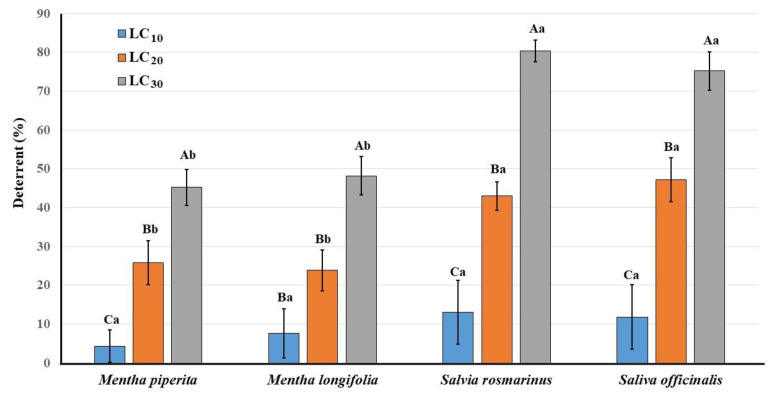
Deterrent effects (%) of the three lethal concentrations of tested four plant essential oils against imago of *Aphis punicae.* Different capital letters (among the lethal concentration of the same EO) and small letter (among EOs of the same lethal concentration) above bars indicate significantly different means according to Tukey’s test (*p* < 0.05). Bars indicate the standard error (SE).

The effect of the tested EOs with three less lethal concentrations (LC_10_, LC_20_, and LC_30_) on the deterrence of *A. punicae* is presented in Figure 1. In the control treatment, the average nymph production was 18.6 nymphs/5 adults/3 days. The lowest deterrence was achieved with LC_10_ of *M. piperita* (4.3%), while the highest values were achieved with LC_30_ of *S. officinalis* (80.4%) and *S. rosmarinus* (75.2%). There were significant differences among the deterrent effect of the three tested LCs for all EOs on *A. punicae* (F_2,12_ = 16.76, *p* < 0.001 for *M. piperita*; F_2,12_= 13.69, *p* < 0.001 for *M. longifolia*; F_2,12_ = 38.85, *p* < 0.001 for *S. officinalis* and F_2,12_= 24.11, *p* < 0.001 for *S. rosmarinus*). Meanwhile, LC_10_ indicated that there was no significant difference among the tested EOs (F_3,16_ = 0.326, *p* = 0.81), while LC_20_ (F_3,16_ = 5.35, *p* = 0.01) and LC_30_ (F_3,16_ = 16.57, *p* < 0.001) indicated a significant difference between both *Menth* sp. and both *Salvia* sp. In general, Two-Way ANOVA analyses (Table 4) showed that there was a significant difference among the deterrent effect of the tested EOs on *A. punicae* (F = 11.779, df = 3, *p* < 0.001). Moreover, there was a significant difference in the deterrent effect among the tested concentrations (F = 88.948, df = 2, *p* < 0.001). Meanwhile, the interaction effect between the tested concentrations and the tested EOs showed no significant difference (F= 1.77, df= 6, *p* = 0.125).

### 3.4. Repellent Activity

**Figure 2:**
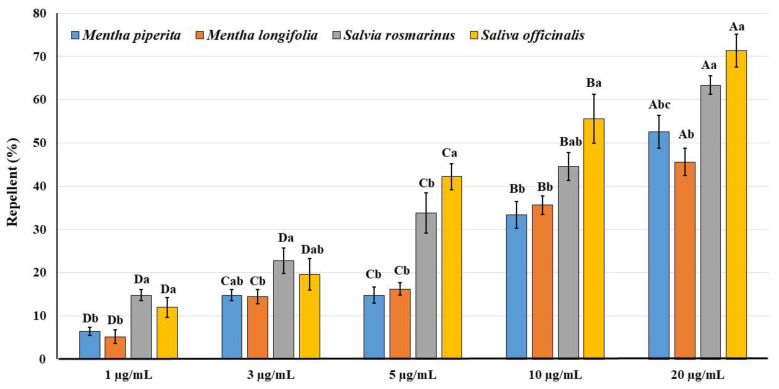
Repellent effects (%) of five concentrations of the tested four plant essential oils against imago of *Aphis punicae.* Different capital letters (among the concentrations of the same EO) and small letter (among EOs of the same concentration) above bars indicate significantly different means according to Tukey’s test (*p* < 0.05). Bars indicate the standard error (SE).

The repellence of the tested EOs at five different concentrations compared with the control is shown in Figure 2. The repellence of the tested EOs increased with the concentration. The lowest repellence was achieved with 1 µg/mL of both *M. piperita* (6.4%) and *M. longifolia* (5.2%), while the highest repellence was achieved with the highest concentration of 20 µg/mL for *S. officinalis* (63.4%) and *S. rosmarinus* (71.4%). There were significant differences in the repellent effect of the five tested concentrations for all EOs on *A. punicae* (F_4,20_= 57.38, *p* < 0.001 for *M. piperita*; F_4,20_= 64.41, *p* < 0.001 for *M. longifolia*; F_4,20_= 38.53, *p* < 0.001 for *S. officinalis* and F_4,20_= 41.05, *p* < 0.001 for *S. rosmarinus*).

## 4. Discussion

The EO yields for the tested plants ranged from 0.72 to 1.35 (w/w). Moreover, there were variations in the major EO constituents’ content for all tested plant species. In general, the environmental conditions (especially altitude) and collection site affected the EO yields and chemical compositions, where altitude is considered an indirect control affecting plant metabolism [26,27]. Moreover, different species of the same genus differed in terms of both EO yield and their components. For example, different species of *Salvia* sp. collected from the same region had different yields and chemical compositions [28,29]. Additionally, for *Mentha* sp., the same results were obtained [30]. In a previous investigation, the EO yield of *S. officinalis* (1.72–2.06%) was higher than that of *S. rosmarinus* (0.99–1.08%), which were collected from the same region and at the same altitude [27]. This result is in line with our results for these two species. Moreover, three different species of *Mentha* sp. varied in their yield of EOs: *Mentha pulegium* (2.93%), *M. piperita* (1.23%) and *Mentha spicata* (0.9%) [31]. Biorational insecticides such as EOs are suitable for replacing the synthetic insecticide functions in integrated pest management programs. The positive attributes usually associated with them include higher specificity and safety to non-target, low environmental and mammalian risk, lower risk of resistance development, and lower environmental persistence [32,33,34].

Many investigations demonstrate that EOs repel the insects and also act on them as neurotoxic compounds [35,36] where some of their components inhibit the activity of acetylcholinesterases (AChE) [37] such as α-pinene and β-pinene, phellandrene, limonene, menthol, menthone, and Carvone, those obtained in this study. EO components such as pulegone have an effect on gamma-amminobutyric acid (GABA) [38] and on octopamine receptors of different insects and can exhibit antagonistic or synergistic activity [39]. These effects indicate that there are diverse mechanisms of action of EO components [40].

Our findings revealed that both the *S. officinalis* and *S. rosmarinus* EOs were more toxic than both *M. piperita* and *M. longifolia* for aphids and the coccinellid predator. In this regard, the effects of the EO compounds, rosacide (from *S. rosemarinus*), sagix (from *S. officinalis*), and cura (from *Curcuma longa*), on *Aphis craccivora* were very promising, especially at higher concentrations [41], and they also exerted a moderate impact on the predator, *Rodolia cardinalis* larvae. However, *S. officinalis* achieved 100% mortality for *Acyrthosiphon pisum*, but caused 45% mortality for *Myzus persicae* with the same concentration (2 µL/L) through the fumigation method [42]. On other insect pests, rosemary EO showed a strong impact on *Trichoplusia ni* due to the increased penetration of the tested toxicants through the integument rather than through the inhibition of detoxicative enzymes [43].

In the present study, the effects of the EOs varied according to the plant species and concentration, where the mortality rates increased with an increase in the EO concentration for all EOs on both *A. punicae* and *C. undecimpunctata*. This finding is in agreement with many previous investigations [44,45,46]. Moreover, all tested concentrations for all tested EOs had the highest toxicity for *A. punicae* compared to *C. undecimpunctata.* In general, the EOs were more effective towards target insect pests than natural enemies. Regarding the contact toxicity, *Satureja intermedia* EO was more toxic for imago females of *Aphis nerii* than *Coccinella septempunctata* imago [46]. Other investigations stated that insect predators are more tolerant to various EOs than aphids [30,47,48]. A previous investigation reported that with four aphid species tested, the most effective EOs from the four tested EOs were in some cases up to five times more toxic than that of the coccinellid predators [29]. Computational docking analysis reinforced such selectivity actions as the Negramina essential oil major compounds bound to the TRP channels of *Myzus persicae* but not to ladybeetle-related TRP channels. Interestingly, the exposure to the Negramina essential oil did not affect the predatory abilities of *Coleomegilla maculata* but increased the abilities of *Eriopis connexa* to prey upon *M. persicae*. These findings provided a physiological basis for the insecticidal and selectivity potential of this EO. Such differential susceptibility results from the differences in the life history traits and in differential receptor expressions (quantities and/or types) in aphids and ladybeetles [49]. 

The repellent activity of EOs from various plants has been clearly confirmed through their major active constituents [50]. In this study, the repellence (%) of the EOs increased with the concentration, where the highest repellence was achieved with the higher concentration (20 mg/mL) of *S. officinalis* (63.4%) and *S. rosmarinus* (71.4%). This result is in accordance with previous findings [50], where rosemary EO had a repellent effect against the onion aphid *Neotoxoptera formosana* and may play a role in plant defense against attacks by insect pests. The repellent activity of *S. rosmarinus* EOs against female imago of *Planococcus citri* under laboratory conditions amounted to 57.1 and 45.2% in choice and no-choice tests, respectively [51]. Also, it had a high negative impact against adults of *Acanthoscelides obtectus* and *Leptinotarsa decemlineata* [52]. The repellent efficacy of EOs could provide a potential direct method to protect plants through the application of phytochemical repellents [53].

In the present study, deterrence also increased with LC increase, where the highest values were achieved with LC_30_ of *S. officinalis* (80.4%) and *S. rosmarinus* (75.2%), and there were significant differences among the tested EOs as well as the tested concentrations; however, the interaction effect between the tested concentrations and the tested EOs showed no significant difference. The same findings were obtained previously, where the Two-Way ANOVA revealed that there was a significant difference in the deterrent effect for the cabbage aphid *Brevicoryne brassicae* among different five EOs and also two different concentrations, while the interaction effect of different essential oils at different concentrations showed no significant difference [54]. The deterrent activity of *S. rosmarinus* EOs against female imago of *Planococcus citri* under laboratory conditions amounted to 57.1 and 45.2% in choice and no-choice tests, respectively [51]. Evaluation of the effects of seven plant EOs, including *S. rosmarinus* on the cabbage aphid *B. brassicae*, showed that they reduced reproductivity and led to an increased mortality rate in the aphid population [55]. The deterrence of aphid nymph production varied from 6.18 to 84.83% for three different EOs with LC_10_ and LC_25_ against *Brevicoryne brassicae* L. and the black aphid *Aphis fabae* [22]. In general, EOs as biocompatible pesticides, due to their volatility and very short-term persistence in the environment, can be considered an important alternative to chemical pesticides to control aphids [55].

## 5. Conclusions

The findings of the current study show that the studied EOs, especially *S. officinalis* and *S. rosmarinus*, had strong negative effects on the pomegranate aphid *A. punicae* due to the low impact of these EOs for the insect predator. Moreover, an appropriate composition of EOs can be used to control aphids in integrated pest management programs. Therefore, the results suggest that these EOs may help in the population reduction of *A. punicae* through their toxicity, repellent, and nymph production deterrence effects. Moreover, these findings are promising for future research as they have the potential to be launched on a commercial scale. Other future investigations could be performed on these EOs to evaluate their compatibility with other biocontrol agents such as entomopathogenic fungi and bacteria.

## Figures and Tables

**Table 1:** Chemical composition (%) of essential oils of *M. piperita, M. longifolia, S. officinalis,* and *S. rosmarinus*.

R.T.	Compound	Area (%)				Formula
		*Mentha* *piperita*	*Mentha* *longifolia*	*Salvia* *officinalis*	*Salvia* *rosmarinus*	
3.32	α-Phellandrene	3.46				C10H16
3.44	3-Thujene			1.97	1.00	C10H16
3.63	α-pinene	2.05	1.43	12.26	30.09	C10H16
4.00	Camphene			14.44	6.80	C10H16
4.05	2,4(10)-Thujadiene			-	2.06	C10H14
4.08	3-Octanol	0.27				C8H18O
4.47	α-Phellandrene		0.60			C10H16
4.49	α-Phellandrene			1.74	-	C10H16
4.63	α -Pinene			10.42	6.54	C10H16
4.71	Eucalyptol	2.09				C10H18O
4.79	1-Octen-3-ol			0.65	-	C8H16O
4.82	β-Pinene			5.99	5.21	C10H16
4.97	α-Ocimene	2.34				C10H16
5.59	α-Terpinene			0.43	2.05	C10H16
5.98	D-Limonene	4.08	0.75	3.71	5.23	C10H16
6.08	p-Cymene			-	7.08	C10H14
6.10	o-Cymene			0.75	-	C10H14
6.45	Eucalyptol		4.50	33.52	6.91	C10H18O
6.84	γ-Terpinene			1.14	6.01	C10H16
7.07	α-Ocimene			0.23	-	C10H16
7.70	α-Terpinolene				2.65	C10H16
8.35	Linalool			-	1.06	C10H18O
9.46	endo-Borneol	0.50				C10H18O
9.90	Sabinyl acetate		0.15			C10H16O
10.56	(+)-2-Bornanone			11.58	0.62	C10H16O
10.60	l-Menthone		34.49			C10H18O
11.07	Isomenthol		0.35			C10H20O
11.16	endo-Borneol			-	2.55	C10H18O
11.26	Thujone			0.59	-	C10H16O
11.44	dl-Menthol		2.75			C10H20O
11.45	Terpinen-4-ol			-	0.48	C10H18O
12.02	Carveol	1.77				C10H16O
12.14	α-Terpineol			-	0.68	C10H18O
12.20	α-Terpineol		0.77			C10H18O
12.51	Carvone	61.16	3.77			C10H14O
12.67	l-Verbenone			-	0.87	C10H14O
14.05	Pulegone		48.60			C10H16O
14.38	Geraniol			-	2.20	C10H18O
15.55	(-)-Bornyl acetate			-	4.54	C12H20O2
14.64	Piperitone		0.46			C10H16O
15.32	Neocarveol	2.28				C10H18O
16.77	trans-Carveyl acetate	3.64				C12H18O2
17.01	(-)-β-Bourbonene	1.83				C15H24
18.49	Caryophyllene	0.65				C15H24
18.82	trans-Verbenone		0.51			C10H14O
19.17	1-Pentanol, 4-amino-			0.23	-	C5H13NO
19.65	Geranyl acetate			-	1.04	C12H20O2
20.32	(+)-epi-Bicyclosesquiphellandrene	0.58				C15H24
20.83	Caryophyllene			-	1.64	C15H24
20.85	Caryophyllene		0.39			C15H24
21.16	α -Cubebene	10.99				C15H24
22.20	β-Elemen	0.97				C15H24
22.34	Humulene			-	0.90	C15H24
22.88	trans-calamenene				0.46	C15H22
26.81	Cubenol				0.22	C15H26O
27.48	Caryophyllene oxide			-	0.50	C15H24O
28.00	ç-Muurolene	0.38				C15H24
**Total**		** 99.04 **	** 99.52 **	** 99.65 **	** 99.39 **	
	**Number of compounds**	**17**	**14**	**16**	**26**	

R.T., Retention time. -, not detected.

**Table 2:** LC_50_ values (µg/mL) for the tested four plant essential oils against imago of *Aphis punicae* and 3rd instar of *Coccinella undecimpunctata* larvae.

Tested Insect	Essential Oil	LC_50_ (Confidence Interval Limits)	S.R. *	Intercept ± SE	Slope ± SE	ꭓ^2^	*P*
*Aphis punicae*	*Mentha piperita*	2.971 (2.376–3.504)		−0.782 ± 0.126	0.263 ± 0.029	0.675	0.879
	*Mentha longifolia*	2.400 (1.750–2.945)		−0.642 ± 0.127	0.268 ± 0.030	4.443	0.217
	*Salvia officinalis*	1.574 (0.940–2.043)		−0.563 ± 0.151	0.358 ± 0.047	1.001	0.801
	*Salvia rosmarinus*	1.653 (0.945–2.190)		−0.497 ± 0.136	0.301 ± 0.037	6.046	0.109
*Coccinella undecimpunctata*	*Mentha piperita*	10.334 (6.513–16.716)	3.478	−0.970 ± 0.095	0.094 ± 0.009	9.898	0.019
	*Mentha longifolia*	8.737 (3.862–15.949)	3.640	−0.840 ± 0.094	0.096 ± 0.010	13.583	0.004
	*Salvia officinalis*	6.237 (5.452–7.076)	3.963	−1.022 ± 0.106	0.164 ± 0.015	2.036	0.565
	*Salvia rosmarinus*	5.960 (1.764–14.809)	3.606	−0.877 ± 0.100	0.147 ± 0.013	26.640	0.001

* S.R. = selectivity ratio (LC50 for predator/LC50 for aphid).

**Table 3:** Relative susceptibilities of *Aphis punicae* imago and 3rd instar of *Coccinella undecimpunctata* larvae to the tested four plant essential oils.

Plant Extract	*Mentha piperita*	*Mentha* *longifolia*	*Salvia* *officinalis*	*Salvia* *rosmarinus*
*Mentha piperita*		−0.933	**−2.728**	**−3.484**
*Mentha longifolia*	0.538		**−1.795**	**−2.551**
*Salvia officinalis*	**1.705**	1.166		0.756
*Salvia rosmarinus*	**1.509**	0.971	**−0.195**	

RMP values of the comparisons: *Aphis punicae* (lower left of the table), *Coccinella undecimpunctata* (upper right of the table). Values indicate the comparison of EO in the column versus EO in the row; Value > 1 indicates less susceptibility while value < 1 indicates more susceptibility. Bold value indicates significant value (95% CI ≠ 1).

**Table 4:** Two-Way ANOVA for deterrent activity of the tested four plant essential oils on *A. punicae*.

Source	Type III Sum of Squares	Df	Mean Square	F	Sig.
Corrected Model	35,456.85	11	3223.35	20.35	<0.001
Intercept	75,437.60	1	75,437.60	476.27	<0.001
Plant species	5597.15	3	1865.72	11.78	<0.001
Concentration	28,177.31	2	14,088.65	88.95	<0.001
Plant species x Concentration	1682.39	6	280.40	1.77	0.125
Error	7602.80	48	158.39		
Total	11,8497.25	60			
Corrected Total	43,059.65	59			

## Data Availability

All data are available in all figures and tables of the manuscript.

## Supplementary Materials

The following are available online at https://www.mdpi.com/article/10.3390/plants11030463/s1, Figure S1. GC–MS chromatograms of essential oils of *Mentha piperita* (A), *Mentha longifolia* (B), *Salvia officinalis* (C), and *Salvia rosmarinus* (D).
