# Supplementary figures and images for: Toxicity, Deterrent and Repellent Activities of Four Essential Oils on Aphis punicae (Hemiptera: Aphididae)
Source: Plants (Basel). 2022 Feb 8;11(3):463. doi: 10.3390/plants11030463 (PMC8839614; doi:10.3390/plants11030463)

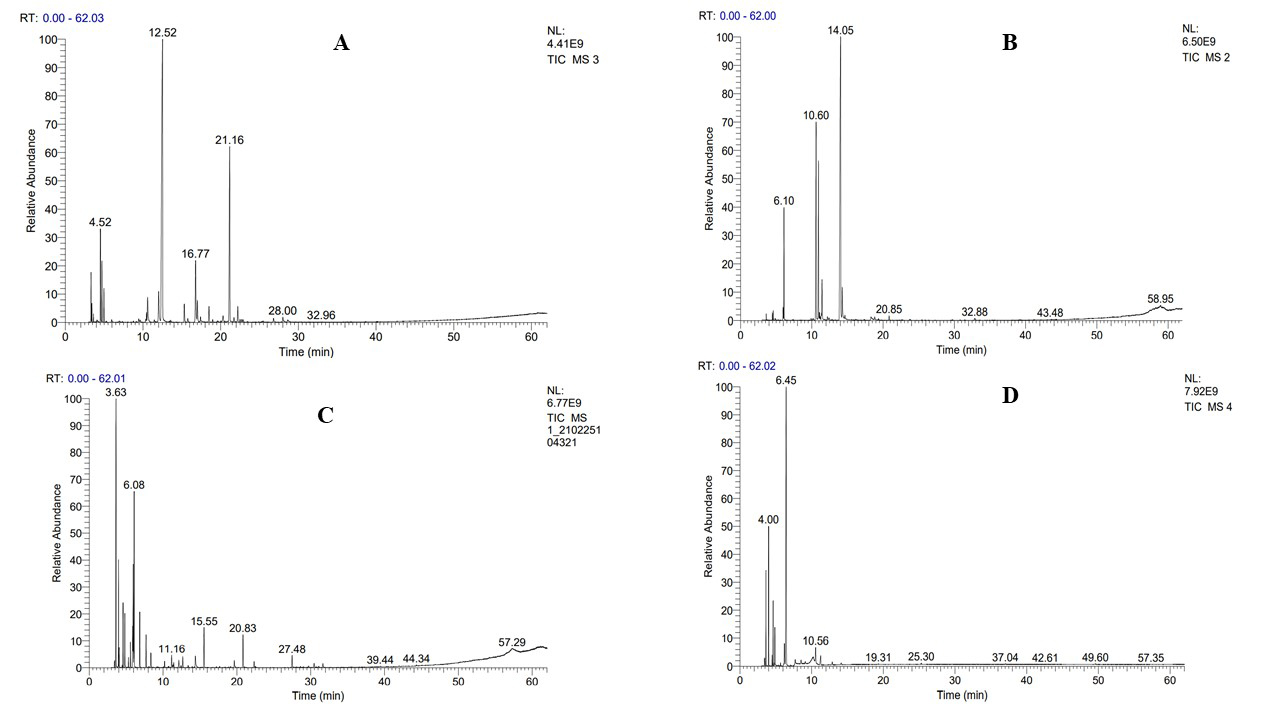

Supplement: Supplementary file 1 [file plants-11-00463-s001.zip › plants-1571889-supplementary.jpg]
